# AI-Assisted Multimodal Transcriptomic Analysis Identifies a Senescence-Related Prognostic Signature and Characterizes ADGRF5-Associated Malignant Phenotypes in Breast Cancer

**DOI:** 10.3390/cells15171589

**Published:** 2026-09-01

**Authors:** Wenhao Liu, Wenhui Wu, Shubai Chen, Kaiqiong Chen, Xin Li

**Affiliations:** 1 College of Life Science, Northeast Agricultural University, Harbin 150030, China; 2 State Key Laboratory of Organ Regeneration and Reconstruction, Institute of Zoology, Chinese Academy of Sciences, Beijing 100101, China; 3 Beijing Institute for Stem Cell and Regenerative Medicine, Beijing 100101, China; 4 Institute of Computing Technology, Chinese Academy of Sciences, Beijing 100101, China; 5 School of Medicine, Xiamen University, Xiamen 361102, China

**Keywords:** cellular senescence, breast cancer, artificial intelligence, prognostic signature

## Abstract

Cellular senescence (CS) is increasingly recognized as an important cell-state programme involved in breast cancer progression and therapeutic response, but its context-dependent molecular heterogeneity limits its application in prognostic assessment. In this study, GeneCompass-based all-gene in silico perturbation analysis was performed to identify candidate genes predicted to induce senescence or rejuvenation, thereby expanding the known senescence-related gene set. Machine learning further established a seven-gene prognostic signature that may serve as an adjunctive tool for prognostic assessment across multiple cohorts. The time-dependent AUCs at 1, 3, and 5 years were 0.707, 0.700, and 0.684 in the training cohort; 0.657, 0.661, and 0.629 in the test cohort; and 0.611, 0.646, and 0.637 in the external validation cohort, respectively. Single-cell and spatial transcriptomic analyses suggested that the risk component of the prognostic signature reflects not only malignant epithelial cell states but also stromal–vascular remodelling in the tumor microenvironment. Among the signature genes, ADGRF5 exhibited the most pronounced expression alteration, and its knockdown suppressed malignant phenotypes in breast cancer cells. These findings provide an AI-assisted strategy for senescence biomarker discovery and highlight ADGRF5 as a candidate functional risk gene associated with breast cancer progression.

## 1. Background

Breast cancer (BC) is one of the most common malignancies in women and remains a major cause of cancer-related mortality worldwide [1,2,3]. Cancer is characterized by substantial molecular and cellular heterogeneity, which contributes to differences in tumor behavior, therapeutic response, and clinical outcome. Advances in understanding cancer biology have progressively transformed treatment strategies from conventional surgery, radiotherapy, and chemotherapy toward more precise approaches, including endocrine therapy, targeted therapy, and immunotherapy [4]. Despite major advances in breast cancer diagnosis and treatment, clinical outcomes remain highly heterogeneous. [1,5]. Patients with similar clinicopathological characteristics may experience markedly different risks of recurrence, metastasis and death, suggesting that conventional clinical parameters are insufficient to fully capture the biological complexity of BC [5]. Therefore, identifying robust molecular biomarkers and developing reliable prognostic models are essential for improving risk stratification and guiding individualized treatment.

Cellular senescence (CS) is a stable and generally irreversible growth-arrest state induced by multiple stress signals, including DNA damage, oncogene activation, oxidative stress, telomere dysfunction and therapeutic pressure [6,7,8,9,10,11]. Replication stress and persistent DNA damage are closely linked to senescence induction, particularly during oncogene-driven tumorigenesis, where aberrant proliferation can activate DNA damage responses and promote senescence as an early protective barrier [12]. CS was initially considered a tumor-suppressive mechanism because it prevents the uncontrolled proliferation of damaged or premalignant cells [8,9]. However, senescence is increasingly recognized as a dynamic and context-dependent process with both tumor-suppressive and tumor-promoting effects. However, senescent cells can also promote tumor progression through the senescence-associated secretory phenotype (SASP), which reshapes the tumor microenvironment by regulating inflammation, immune infiltration, extracellular matrix remodeling, angiogenesis and epithelial–mesenchymal transition [13,14,15,16]. In BC, senescence-related programs may participate in tumor evolution, treatment resistance, immune remodeling and disease progression [17,18,19]. Nevertheless, the prognostic relevance of CS in BC remains incompletely understood because senescence lacks universal molecular markers and may display distinct features across different cellular contexts [8,9].

Previous studies have mainly investigated senescence-related genes using prior biological knowledge or literature-curated databases. Although these gene sets provide valuable references, they may not fully capture tumor-specific or cell-state-dependent senescence regulators. The rapid development of single-cell foundation models has provided a new opportunity to infer gene function by modeling cell-state transitions at high resolution [20,21,22,23,24]. Models such as Geneformer [21], scGPT [20] and GeneCompass [22] learn high-dimensional representations of genes and cellular states from large-scale single-cell transcriptomic data, enabling in silico perturbation analysis beyond existing annotations. This strategy may help identify previously underappreciated genes involved in senescence-like or rejuvenation-like cellular transitions [25,26].

In this study, we developed an AI-assisted senescence gene discovery framework for BC. Young and old cell single-cell RNA-seq profiles were used as reference states, and GeneCompass-based all-gene perturbation was performed to identify senescence-inducing and rejuvenation-associated genes. These AI-predicted genes were integrated with literature-derived senescence genes and breast cancer differentially expressed genes to construct an expanded senescence-related candidate gene set. Based on this gene set, we identified senescence-related molecular subtypes, constructed and validated a seven-gene prognostic signature, and further explored its cellular and spatial basis using single-cell and spatial transcriptomic analyses. Finally, ADGRF5 was prioritized as a key risk gene and experimentally validated for its role in breast cancer cell migration and invasion.

## 2. Methods

### 2.1. Data Collection

We obtained 1194 independent breast cancer samples from The Cancer Genome Atlas (TCGA) (http://tcga.cancer.gov/ (accessed on 8 October 2025)) database and 459 normal breast tissue samples from the Genotype-Tissue Expression (GTEx) database. In addition, five microarray datasets were downloaded from the Gene Expression Omnibus (GEO) (https://www.ncbi.nlm.nih.gov/geo/ (accessed on 8 October 2025)) database, including GSE58644 containing 314 samples, GSE86166 containing 366 samples, GSE48390 containing 81 samples, GSE42568 containing 104 samples and GSE199633 containing 637 samples.

For single-cell transcriptomic analysis, two publicly available single-cell RNA-seq datasets were collected, including E-MTAB-13664 from the European Bioinformatics Institute (EBI) database and GSE180286 from the GEO database. Among these, E-MTAB-13664 was specifically used to construct the young- and aged-donor reference states for the AI-assisted analysis. Only normal human breast tissue donors were included for this purpose. Donors aged ≥ 50 years were classified as aged donors, comprising 23 donors and 220,245 cells, whereas donors aged ≤ 30 years were classified as young donors, comprising 44 donors and 346,503 cells. These age-stratified profiles were subsequently used as reference states for the AI-assisted identification of senescence-associated candidate genes. The GSE180286 scRNA-seq dataset included five patient samples. After quality control, a total of 26,770 cells were retained for subsequent single-cell transcriptomic analyses. The GSE180286 data were subsequently used for Scissor analysis. Furthermore, three spatial transcriptomic samples from GSE203612 were included to investigate the gene spatial distribution of senescence-related malignant cell populations in breast cancer.

### 2.2. Data Processing

For cross-dataset integration, including TCGA-BRCA with 459 GTEx normal breast samples and the combined external validation cohort consisting of GSE86166, GSE48390, GSE42568, and GSE199633, gene identifiers were converted to gene symbols, duplicated genes were collapsed, and genes shared across the corresponding datasets were retained. The expression matrices were subsequently merged based on shared genes, with dataset origin specified as the batch variable. Batch effects were corrected using the ComBat algorithm implemented in the “sva” R package. The batch-corrected expression matrices were used for subsequent comparative and survival analyses.

Single-cell RNA-seq and spatial transcriptomic data were processed using the R package “Seurat”. For scRNA-seq datasets, low-quality cells were removed according to the following criteria: nCount_RNA > 1000, nFeature_RNA > 300, mitochondrial gene percentage < 20%, ribosomal gene percentage > 3% and haemoglobin gene percentage < 0.1%. Principal component analysis was performed for dimensionality reduction, followed by uniform manifold approximation and projection (UMAP) for visualization. Cell clusters were identified using the “FindNeighbors” and “FindClusters” functions, and major cell types were annotated manually based on canonical marker genes.

Spatial transcriptomic data were normalized using the “SCTransform” function in Seurat. Principal component analysis and UMAP were then performed for dimensionality reduction and visualization.

### 2.3. Differentially Expressed Genes (DEGs)

The R package “DESeq2” was used to identify differentially expressed genes (DEGs), and genes meeting the criteria of |log2FC| > 1 and adjusted *p* value < 0.05 were retained for subsequent analyses [27]. GO enrichment analysis was then carried out using the “clusterProfiler” package to explore the biological functions of these genes. Enriched GO terms with an adjusted *p* value < 0.05 were regarded as significant [28].

### 2.4. In Silico Perturbation Using Single-Cell Foundation Models

To identify senescence-inducing and rejuvenation-associated genes, we performed directional in silico perturbation analyses using three single-cell foundation models: Geneformer, scGPT and GeneCompass. Young-donor and Aged-donor single-cell RNA-seq profiles were used as reference states. The young-to-old direction was used to identify senescence-inducing genes, whereas the old-to-young direction was used to identify rejuvenation-associated genes.

For each candidate gene g, a loss-of-function-like perturbation was introduced in the source-state cells and the perturbed cells were re-encoded by the corresponding model. Let $z_{i}^{\left(0\right)}$ denote the baseline embedding of source cell i, $z_{i,g}^{\left(-\right)}$ the embedding after perturbation of TF g, and $c_{T}$ the centroid embedding of the target reference state. The directional effect was calculated as:

(1) $$\Delta_{g}\;=\;\frac{1}{N}\;\sum_{i=1}^{N}\left[cos\left(z_{i,g}^{\left(-\right)},\;c_{T}\right)\;-\;cos\left(z_{i}^{\left(0\right)},\;c_{T}\right)\right]$$

A positive $\Delta_{g}$ indicated that perturbation shifted the source-cell representation toward the target reference state; a negative value indicated a shift away from that state. Genes were ranked in descending order of $\Delta_{g}$, and the top 100 genes in each direction were retained to obtain balanced and comparable candidate sets across perturbation directions and foundation models. Empirical *p* values were calculated against a null distribution and adjusted using the Benjamini–Hochberg method to assess statistical support but were not used as a criterion for gene selection. For each analysis, 1000 cells were randomly sampled from the source state and 1000 cells were randomly sampled from the target state. All experiments were performed three times.

For Geneformer, we used the pretrained Geneformer V1 10M model and its in silico perturbation framework. Single-cell transcriptomes were represented as rank-ordered gene-token sequences. For each candidate gene, the corresponding gene token was removed from the source-cell sequence, and the perturbed sequence was re-encoded by Geneformer. The change in similarity between the baseline or perturbed source-cell embedding and the target-state embedding centroid was then used to calculate the directional perturbation effect.

As the official scGPT implementation does not provide a dedicated in silico perturbation module, we implemented a Geneformer-analogous perturbation workflow in the scGPT representation space. Genes were mapped to the scGPT human vocabulary, and the cell-level CLS embedding was used as the representation. For each candidate gene, its expression value was set to zero in source-state cells that expressed the gene, after which the perturbed cells were re-embedded by scGPT. The directional effect was then calculated using the same target-centroid cosine-similarity framework described above.

For GeneCompass, we implemented a Geneformer-like loss-of-function perturbation design within the GeneCompass representation framework. GeneCompass encodes gene identity and quantitative expression together with prior biological knowledge, including gene-regulatory, promoter, gene-family and co-expression information. For each candidate gene, its expression value was set to zero in the source-state cells before encoding. The baseline and perturbed cells were represented using the special cell token embedding, and the directional effect was calculated using the same target-centroid cosine-similarity metric, $\Delta_{g}$. The sampling strategy, transition directions, candidate gene set, null-model construction and ranking procedure were matched to those used for Geneformer.

### 2.5. Consensus Clustering Based on AI-Expanded Senescence Features

To evaluate whether senescence-related genes expanded by different AI foundation models could define distinct molecular patterns in breast cancer, unsupervised consensus clustering was performed in the TCGA-BRCA cohort using the R package “ConsensusClusterPlus” [29]. For each AI-expanded senescence gene set, the expression matrix of candidate genes was extracted and used for clustering analysis. The clustering procedure was repeated 1000 times to ensure classification stability. The optimal number of clusters was determined according to the cumulative distribution function (CDF) curve and the relative change in the area under the CDF curve.

### 2.6. Mutation Analysis

Somatic mutation profiles of breast cancer patients were downloaded from the TCGA-BRCA cohort in mutation annotation format (MAF). The mutation data were processed and visualized using the R package “maftools” [30]. The overall mutation landscape and frequently mutated genes were compared among different senescence-related molecular subtypes. The total number of somatic mutations was calculated for each patient, and a log2(somatic mutation count + 1) transformation was applied for subsequent comparison among subtypes.

### 2.7. Immune Infiltration Analysis

Immune infiltration characteristics were assessed using ESTIMATE [31] and CIBERSORT [32]. ESTIMATE was used to calculate immune and stromal scores for each breast cancer sample and was further applied in subsequent analyses to characterize the tumor microenvironment. The relative abundance of 22 tumor-infiltrating immune cell types was further inferred by CIBERSORT using the LM22 reference matrix. The resulting immune infiltration features were compared across senescence-related molecular subtypes.

### 2.8. Construction of the Risk Scoring Model

Prognosis-related genes were first identified from senescence-associated candidate genes using Cox regression analysis. To improve the robustness of feature selection, the random forest algorithm was applied to rank candidate genes according to their predictive importance. Genes selected by random forest were then incorporated into LASSO Cox regression analysis using the R package “glmnet” [33]. The optimal value of the penalty parameter was determined by cross-validation, and genes with non-zero coefficients were retained.

A seven-gene risk scoring model was finally established based on TFF1, SUSD3, PTPRT, LYVE1, HBEGF, CD3E and ADGRF5. The risk score of each patient was calculated according to the following formula:

(2) $$Risk\;score=\sum_{i=1}^{n}(\left.Expression_{i}\times Coefficient_{i}\right)$$

Patients were stratified into high- and low-risk groups according to the optimal cut-off of the risk score determined using maximally selected rank statistics with the log-rank criterion. The prognostic value of the model was evaluated using Kaplan–Meier survival curves, Cox regression analysis and time-dependent ROC analysis.

### 2.9. Cell–Cell Communication Analysis

The R package “CellChat” [34] was used to investigate cell–cell communication in single-cell and spatial transcriptomic datasets. Ligand–receptor interactions between malignant cells and other cell types were inferred, and communication probabilities and related signalling pathways were systematically evaluated. Differential communication patterns involving prognosis-associated malignant cell populations were further analysed.

### 2.10. Cell Culture

Human breast cancer cell lines MDA-MB-231 and SUM159, as well as the non-tumorigenic mammary epithelial cell line MCF10A, were used in this study. MDA-MB-231 and SUM159 cells were maintained in DMEM medium (Gibco) supplemented with 10% fetal bovine serum and 1% penicillin–streptomycin. MCF10A cells were cultured in MCF10A complete medium (Procell, CM-0525) according to the manufacturer’s instructions. All cells were incubated at 37 °C in a humidified atmosphere containing 5% CO_2_.

### 2.11. Gene Knockdown

siRNA and shRNA transfection was performed using Lipofectamine 3000 reagent (Invitrogen, Waltham, MA, USA) following the manufacturer’s protocol. Opti-MEM reduced-serum medium was used to prepare the transfection complexes. Cells were seeded to reach approximately 60–70% confluence at the time of transfection.

In SUM159 cells, siRNAs targeting ADGRF5 and HBEGF were synthesized by Tsingke Biotech (Beijing, China). Three independent siRNA duplexes were designed for each gene, named siRNA-1 to siRNA-3, and the final siRNA concentration was 100 nM. In MDA-MB-231 cells, shRNA constructs were also synthesized by Tsingke Biotech, including the negative control shNTC sequence 5′-GTTCTCCGAACGTGTCACGTT-3′ and two shRNAs targeting ADGRF5/GPR116: shGPR116#1, 5′-CGGCTGAAGAATACACTGTTAC-3′, and shGPR116#2, 5′-GGACTACAACTCCTTTCAAGCC-3′ [35]. After 4–6 h of transfection, the medium was replaced with complete culture medium. Cells were collected 48 h after transfection for subsequent experiments.

### 2.12. RNA Extraction

Total RNA was isolated from cultured cells using TRIzol reagent (Invitrogen) according to the manufacturer’s instructions. RNA concentration and purity were measured using a NanoDrop spectrophotometer. Only RNA samples with an A260/A280 ratio between 1.8 and 2.1 were used for further analysis.

### 2.13. Quantitative Real-Time PCR

Complementary DNA was synthesized using a reverse transcription kit from Takara. Quantitative real-time PCR was performed using SYBR Green reagent (Applied Biosystems, Waltham, MA, USA) on a real-time PCR system. Each reaction was performed in a 10 μL reaction volume.

The amplification procedure was as follows: initial denaturation at 95 °C for 2 min, followed by 40 cycles of denaturation at 95 °C for 5 s and annealing/extension at 60 °C for 10 s. Melting curve analysis was performed to confirm amplification specificity. Relative gene expression was calculated using the 2^−ΔΔCt^ method, with GAPDH used as the internal control. Primer sequences are listed in Table S1.

### 2.14. Bulk RNA Sequencing

Total RNA was extracted using TRIzol reagent. RNA quality was assessed using an Agilent 2100 Bioanalyzer, and samples with an RNA integrity number greater than 7.0 were used for library construction. Full-length cDNA libraries were generated using the Smart-seq2 workflow by Tsingke Biotech (China) and subsequently sequenced on an Illumina platform with paired-end 150-bp reads. Three biological replicates were included in each group. Differential expression analysis was conducted using the DESeq2 package. Genes with an absolute log2 fold change greater than 1 and an adjusted *p* value less than 0.05 were considered differentially expressed.

### 2.15. Cell Invasion Assay

Cell invasion ability was evaluated using Corning Matrigel Invasion Chambers (Corning, 354,480, Corning, NY, USA). Briefly, cells suspended in serum-free DMEM were seeded into the upper chambers, while DMEM containing 10% fetal bovine serum was added to the lower chambers as a chemoattractant. After 24 h of incubation, non-invading cells remaining on the upper surface of the membrane were gently removed. Invaded cells on the lower surface were fixed, stained and counted under a microscope. Three independent biological replicates were performed for each experimental group.

### 2.16. Wound Healing Assay

Cells were seeded into 6-well plates and cultured until they reached full confluence. A straight scratch was generated in the cell monolayer using a sterile 200 μL pipette tip. Detached cells were removed by washing with PBS, and the culture medium was replaced with low-serum medium. Images were captured at 0 h and 20 h using an inverted microscope. Wound closure was quantified using ImageJ (version 1.54g) software. Three independent biological replicates were performed for each experimental group.

### 2.17. Senescence-Associated β-Galactosidase Staining

Senescence-associated β-galactosidase (SA-β-Gal) activity was assessed using a Cell Senescence β-Galactosidase Staining Kit (Beyotime Biotechnology, Shanghai, China; Cat. No. C0602; 100 tests) according to the manufacturer’s instructions. Following the indicated treatments, the culture medium was removed, and the cells were gently washed once with phosphate-buffered saline (PBS). The cells were then completely covered with β-galactosidase staining fixative and fixed for 15 min at room temperature. After removal of the fixative, the cells were washed three times with PBS for 3 min each. Fresh β-galactosidase staining working solution was prepared according to the manufacturer’s protocol and added in a sufficient volume to cover the cells. The culture plates were sealed to minimize evaporation and incubated overnight at 37 °C in a CO_2_-free environment protected from light.

After SA-β-Gal staining, the staining solution was removed, and the cells were washed three times with PBS. The cells were subsequently incubated with an appropriate volume of DAPI staining solution for 5 min at room temperature in the dark, followed by three washes with PBS. Images of the same fields were acquired using bright-field and fluorescence microscopy for the detection of SA-β-Gal staining and DAPI-labelled nuclei, respectively. Cells containing blue cytoplasmic precipitates were considered SA-β-Gal-positive, whereas DAPI-positive nuclei were used to determine the total cell number. At least three randomly selected, non-overlapping fields were analyzed for each sample. The percentage of SA-β-Gal-positive cells was calculated as the number of blue-stained cells divided by the number of DAPI-positive nuclei multiplied by 100%.

### 2.18. Statistical Analysis

All statistical analyses were conducted using R (version 4.4.0) software and GraphPad Prism (version 9.0.0). Survival differences were assessed by Kaplan–Meier analysis, with *p* values calculated using the log-rank test. Univariate and multivariate Cox regression analyses were performed to evaluate the prognostic significance of candidate variables. Quantitative experimental data are shown as mean ± SD, and comparisons among multiple groups were conducted using one-way ANOVA with Tukey’s multiple-comparison test. A two-sided *p* value < 0.05 was regarded as statistically significant.

## 3. Results

### 3.1. Identification of AI-Expanded Senescence-Related Genes in Breast Cancer

**Figure 1:**
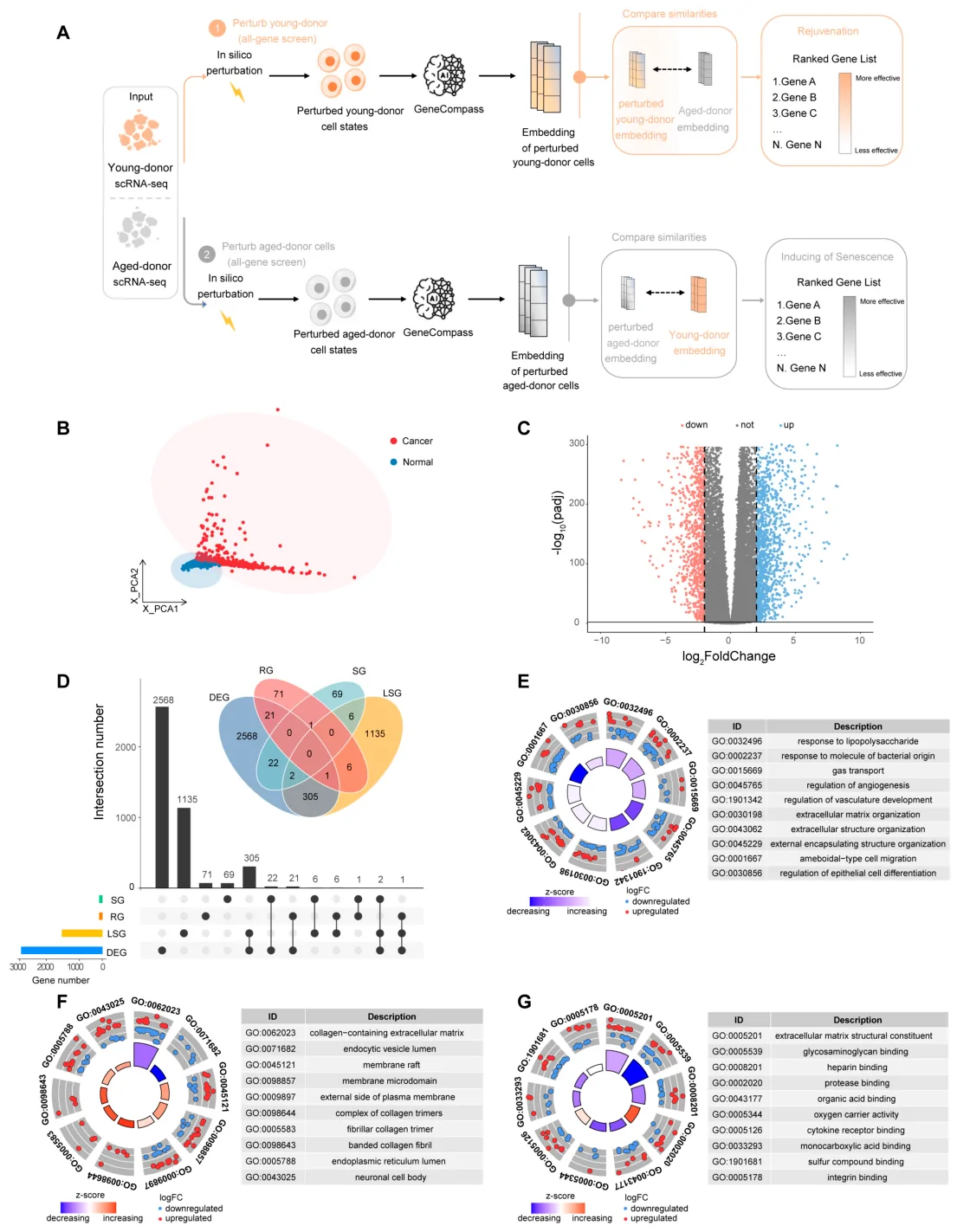
Identification of AI-expanded senescence-related candidate genes in breast cancer. (**A**) Schematic workflow of GeneCompass-based in silico perturbation analysis. (**B**) Principal component analysis of tumor and normal breast tissue samples from the TCGA-BRCA cohort. (**C**) Volcano plot showing differentially expressed genes between breast cancer and normal samples. (**D**) UpSet plot and Venn diagram showing the overlap among breast cancer differentially expressed genes (DEGs), GeneCompass-derived rejuvenation-associated genes (RG), GeneCompass-derived senescence-inducing genes (SG), and literature-derived senescence-related genes (LSG). (**E**–**G**) Gene Ontology enrichment analysis of the candidate genes, including biological process (**E**), cellular component (**F**), and molecular function (**G**).

To identify senescence-related genes with potential relevance to breast cancer, we first applied a GeneCompass-based in silico perturbation strategy using young and old cell single-cell RNA-seq profiles. Through two complementary perturbation directions, genes were ranked according to their ability to shift cellular embeddings towards either young or old reference states. Genes whose perturbation moved old cells towards young-like states were defined as rejuvenation-associated genes, whereas genes whose perturbation moved young cells towards old-like states were defined as senescence-inducing genes. The top 100 genes from each direction were selected for subsequent analysis (Figure 1A).

We then investigated the transcriptomic differences between breast cancer and normal breast tissues in the TCGA-BRCA cohort. Principal component analysis showed a clear separation between tumor and normal samples, indicating distinct global transcriptional profiles between the two groups (Figure 1B). Differential expression analysis further identified extensive gene expression alterations in breast cancer compared with normal tissues (Figure 1C).

To construct a breast cancer-associated senescence candidate gene set, we integrated four gene sources: differentially expressed genes between breast cancer and normal tissues, GeneCompass-derived rejuvenation-associated genes (RG), GeneCompass-derived senescence-inducing genes (SG), and literature-derived senescence-related genes (LSG). UpSet and Venn analyses showed partial overlap among these gene sets, while each gene set also retained distinct components, suggesting that GeneCompass-derived genes provided additional information beyond conventional literature-curated senescence genes. Finally, 351 candidate genes were retained for downstream analyses (Figure 1D).

Functional enrichment analysis was performed to characterize the biological relevance of these candidate genes. GO analysis showed that the genes were mainly enriched in inflammatory responses, angiogenesis regulation, extracellular matrix organization, epithelial cell differentiation, cell migration and membrane-associated signalling components (Figure 1E–G). These processes are closely related to classical senescence-associated features, including senescence-associated inflammatory signalling, extracellular matrix remodelling, vascular microenvironment alteration and epithelial plasticity. These results suggest that the AI-expanded senescence-related gene set captures key senescence-associated biological programmes in breast cancer and may be involved in tumor progression and prognosis.

### 3.2. AI-Expanded Senescence Genes Define Prognostically Distinct Breast Cancer Subtypes

**Figure 2:**
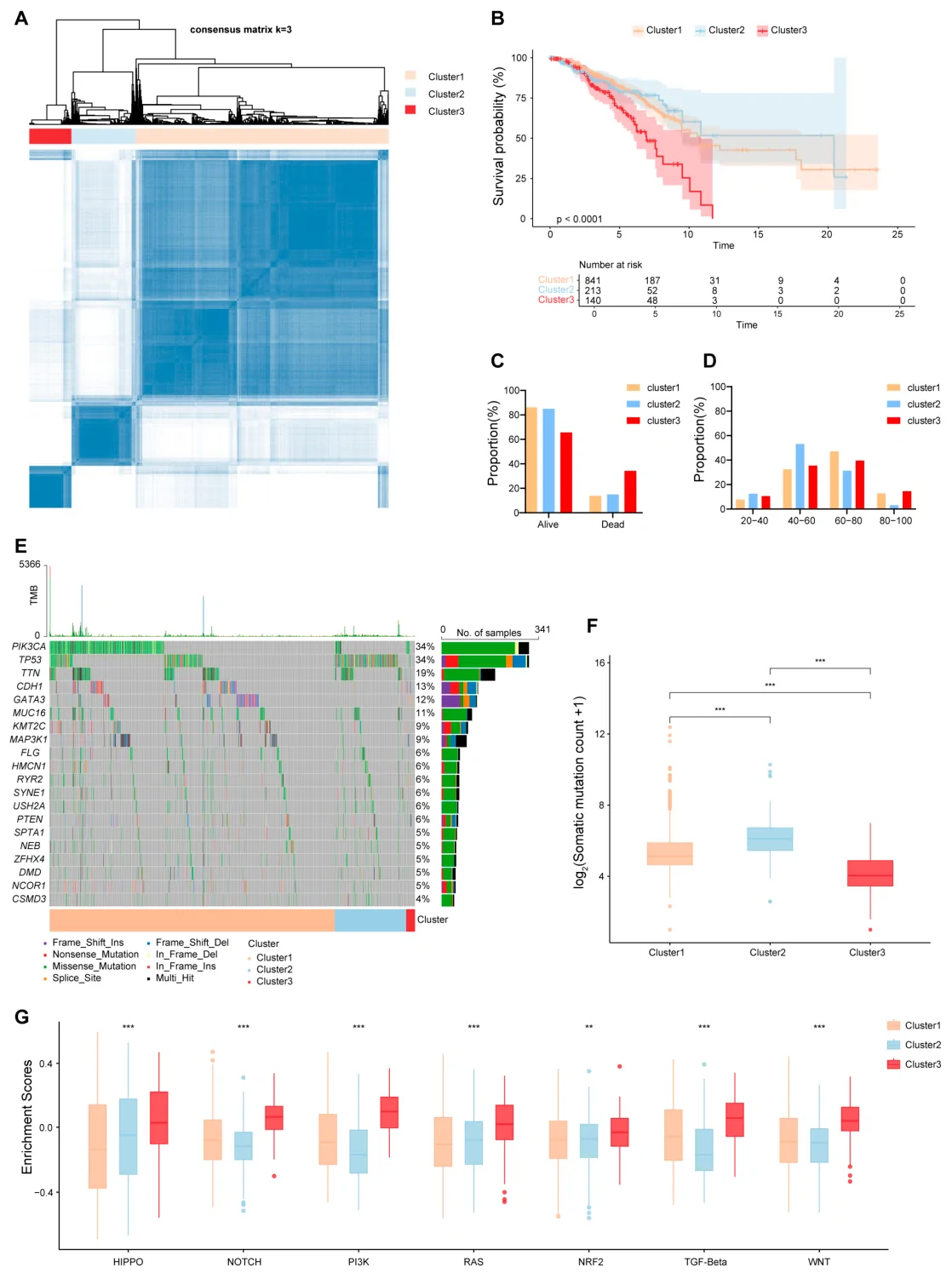
Prognostic and molecular characteristics of senescence-related breast cancer clusters. (**A**) Consensus clustering matrix of breast cancer patients based on the 351 AI-expanded senescence-related genes, with k = 3. (**B**) Kaplan–Meier survival analysis of the three senescence-related clusters. (**C**,**D**) Distribution of survival status and age among the three clusters. (**E**) Somatic mutation landscape of the three clusters in the TCGA-BRCA cohort. (**F**) Comparison of log2(somatic mutation count + 1) among the three clusters. (**G**) Enrichment scores of cancer-related signalling pathways across the three clusters. ** *p* < 0.01, *** *p* < 0.001.

To determine whether the 351 AI-expanded senescence-related genes had prognostic relevance in breast cancer, we performed unsupervised consensus clustering in the TCGA-BRCA cohort. Based on the consensus matrix, patients were classified into three senescence-related clusters when k = 3, indicating that these genes could distinguish stable molecular subtypes of breast cancer (Figure 2A). Kaplan–Meier survival analysis showed significant survival differences among the three clusters, with Cluster 3 exhibiting the poorest prognosis (Figure 2B). Consistently, metadata analysis showed that Cluster 3 contained the highest proportion of deceased patients (Figure 2C). In contrast, the age distribution was not markedly skewed towards older patients in Cluster 3, suggesting that the prognostic differences among the clusters were not simply attributable to chronological age, but may instead reflect distinct senescence-related biological states (Figure 2D) [36,37,38,39].

To further evaluate the contribution of AI-assisted gene expansion, we compared consensus clustering results generated from different senescence gene sets. Clustering based only on literature-derived senescence genes, Geneformer-augmented genes, and scGPT-augmented genes all stratified patients into subgroups with significant survival differences (Figure S1A–C). However, comparison of the survival-associated significance levels showed that all AI-augmented gene sets outperformed the literature-only gene set, among which the GeneCompass-augmented gene set showed the strongest prognostic discrimination (Figure S1D). Therefore, the GeneCompass-expanded senescence gene set was selected for subsequent analyses.

We next characterized the genomic features of the GeneCompass-defined clusters. Mutation landscape analysis showed that common breast cancer driver genes, including PIK3CA, TP53, TTN, CDH1 and GATA3, were frequently altered across the cohort (Figure 2E). Notably, tumour mutation burden differed significantly among the three clusters, with Cluster 2 showing the highest log2 (somatic mutation count + 1), whereas Cluster 3 exhibited the lowest level (Figure 2F). A similar pattern was observed for the number of mutated genes, with Cluster 3 displaying the lowest log2-transformed mutation gene count (Figure S1E). These findings suggest that the poor prognosis of Cluster 3 may not be driven by a higher mutational burden [25,40].

Finally, we compared cancer-related pathway activity among the three clusters. Cluster 3 showed significantly higher enrichment scores for multiple oncogenic pathways, including Hippo, Notch, PI3K, RAS, NRF2, TGF-β and Wnt signalling (Figure 2G) [41]. These pathways are closely associated with tumour proliferation, stemness, epithelial–mesenchymal transition, microenvironment remodelling and metastatic progression. Together, these results indicate that AI-expanded senescence-related genes can identify biologically and clinically distinct breast cancer subtypes, with Cluster 3 representing a high-risk senescence-associated subtype characterized by poor prognosis, low mutation burden and broad activation of oncogenic signalling pathways.

### 3.3. Immune Infiltration Characteristics of Senescence-Related Clusters

**Figure 3:**
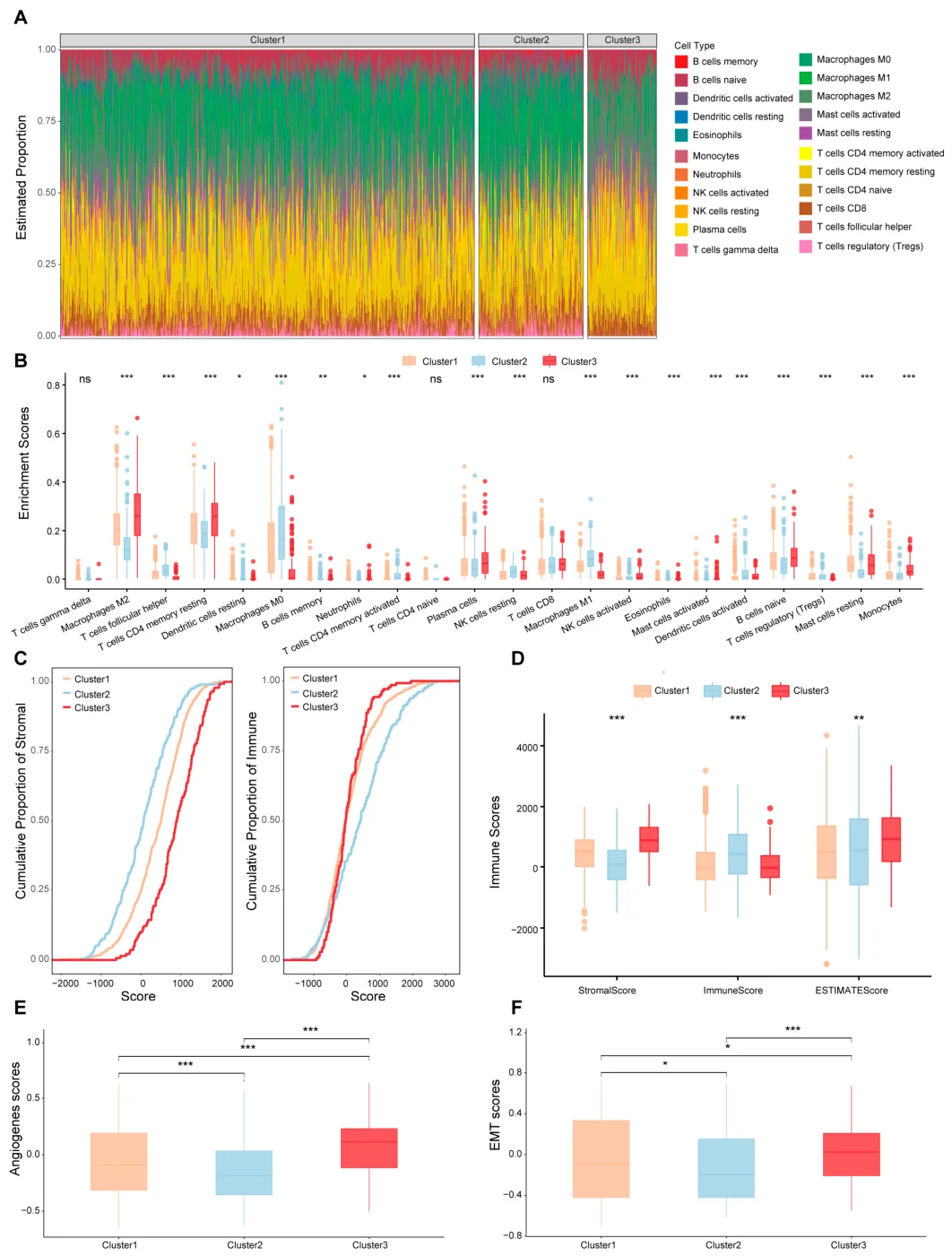
Immune infiltration and tumor microenvironment characteristics of senescence-related breast cancer clusters. (**A**) Relative proportions of 22 immune cell types estimated by CIBERSORT across the three senescence-related clusters. (**B**) Comparison of immune cell enrichment scores among Cluster 1, Cluster 2 and Cluster 3. (**C**) Cumulative distribution curves of stromal and immune scores among the three clusters. (**D**) Comparison of stromal score, immune score and ESTIMATE score among the three clusters. (**E**,**F**) Comparison of angiogenesis scores and EMT scores among the three clusters. ns, not significant; * *p* < 0.05, ** *p* < 0.01, *** *p* < 0.001.

To further characterize the tumour microenvironment of the three senescence-related clusters, we evaluated immune infiltration using CIBERSORT and ESTIMATE. CIBERSORT analysis showed distinct immune cell compositions among the three clusters, indicating that senescence-related subtypes were associated with different immune microenvironmental states (Figure 3A). Multiple immune cell populations differed significantly across clusters, including macrophage subsets, T cell subsets, NK cells, dendritic cells, B cells, mast cells and monocytes (Figure 3B). Notably, Cluster 3 showed relatively higher enrichment of M2 macrophages and resting CD4 memory T cells, whereas several immune effector or activated cell populations were relatively reduced, suggesting an immunologically remodelled and potentially suppressive microenvironment.

ESTIMATE analysis further revealed significant differences in stromal and immune components among the clusters (Figure 3C,D). Cluster 3 displayed the highest stromal score and overall ESTIMATE score, while Cluster 2 showed a relatively higher immune score. These results suggest that the poor-prognosis Cluster 3 is characterized more prominently by stromal activation rather than simple immune enrichment. Consistently, Cluster 3 also exhibited significantly higher angiogenesis and EMT scores than the other clusters (Figure 3E,F), indicating enhanced vascular remodelling and invasive potential. Together, these findings suggest that the high-risk senescence-related subtype is associated with a stromal-rich, angiogenic and EMT-activated tumor microenvironment, which may contribute to its poor clinical outcome.

### 3.4. Construction of a Senescence-Related Prognostic Signature

To develop an adjunctive prognostic model based on the AI-expanded senescence-related gene set, we applied a two-step machine learning strategy to the 351 candidate genes. Random forest analysis was first used to evaluate the predictive importance of each gene. The model error gradually stabilized as the number of trees increased, indicating that the random forest model was suitable for feature ranking (Figure S2A). Candidate genes with higher variable importance were then retained for further screening (Figure S2B). Subsequently, LASSO Cox regression was performed to reduce feature redundancy and avoid overfitting. The coefficient profile and cross-validation curve identified the optimal penalty parameter, and seven genes were finally selected for model construction, including ADGRF5, CD3E, HBEGF, LYVE1, PTPRT, SUSD3 and TFF1 (Figure S2C,D).

The risk score for each patient was calculated as follows:

(3) $$\begin{array}{ll} Risk\;score= & 0.05982\times Expression_{ADGRF5}-0.38594\times Expression_{CD3E}\\ & +0.11608\times Expression_{HBEGF}+0.09381\times Expression_{LYVE1}\\ & -0.17346\times Expression_{PTPRT}-0.11654\times Expression_{SUSD3}\\ & -0.35157\times Expression_{TFF1} \end{array}$$

where Expression represents the normalized expression value of each gene. According to the coefficient direction, ADGRF5, HBEGF and LYVE1 contributed positively to the risk score, whereas CD3E, PTPRT, SUSD3 and TFF1 contributed negatively, indicating that the model integrates both risk-associated and protective expression signals.

**Figure 4:**
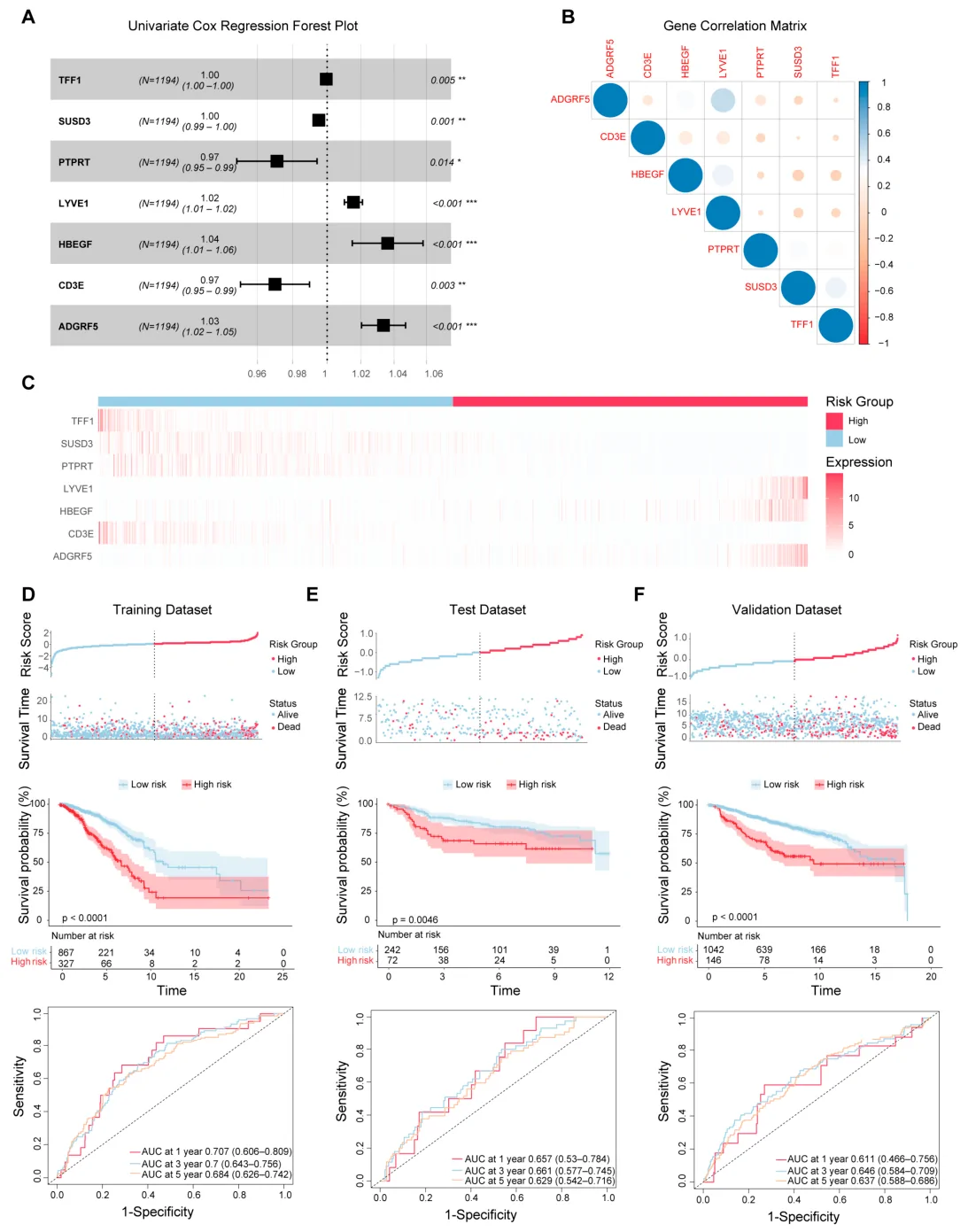
Construction and validation of the seven-gene senescence-related prognostic signature. (**A**) Univariate Cox regression forest plot of the seven signature genes in the TCGA-BRCA cohort. (**B**) Correlation matrix of the seven genes included in the prognostic model. (**C**) Expression distribution of the seven signature genes between the high- and low-risk groups in the TCGA-BRCA cohort. (**D**–**F**) Risk score distribution, survival status, Kaplan–Meier survival analysis and time-dependent ROC curves in the training cohort TCGA-BRCA (**D**), test cohort GSE58644 (**E**), and external validation cohorts including GSE86166, GSE48390, GSE42568 and GSE199633 (**F**). * *p* < 0.05, ** *p* < 0.01, *** *p* < 0.001.

We next evaluated the prognostic relevance and expression characteristics of the seven signature genes. Univariate Cox regression analysis showed that all seven genes were significantly associated with patient survival in the TCGA-BRCA cohort (Figure 4A). Multivariable Cox regression using standardized gene expression showed that CD3E and TFF1 remained significantly associated with survival after mutual adjustment, whereas the associations of the other genes were attenuated (Table S2). Gene correlation analysis showed weak to moderate correlations among most model genes, suggesting limited redundancy and complementary prognostic information (Figure 4B). The expression heatmap further demonstrated distinct expression patterns between the high- and low-risk groups. Genes with positive coefficients, including ADGRF5, HBEGF and LYVE1, were relatively enriched in the high-risk group, whereas genes with negative coefficients, including CD3E, PTPRT, SUSD3 and TFF1, were relatively enriched in the low-risk group (Figure 4C).

Patients were then divided into high- and low-risk groups according to the median risk score. In the TCGA training cohort, patients in the high-risk group had significantly poorer survival than those in the low-risk group (*p* < 0.0001), and the model achieved AUC values of 0.707, 0.700 and 0.684 for 1-, 3- and 5-year survival prediction, respectively (Figure 4D). In the independent test cohort GSE58644, the high-risk group also showed worse prognosis (*p* = 0.0046), with 1-, 3- and 5-year AUC values of 0.657, 0.661 and 0.629, respectively (Figure 4E). Consistently, in the external validation cohorts, including GSE86166, GSE48390, GSE42568 and GSE199633, the risk model remained significantly associated with survival outcome (*p* < 0.0001), with AUC values of 0.611, 0.646 and 0.637 for 1-, 3- and 5-year prediction, respectively (Figure 4F). In addition, Kaplan–Meier survival and time-dependent ROC analyses were performed separately for GSE86166, GSE48390, GSE42568 and GSE199633, further demonstrating the cohort-specific prognostic performance of the model (Figure S3). However, the 1-year AUC estimates were relatively variable across cohorts, and the 95% confidence interval of the pooled 1-year AUC included 0.5, indicating limited discriminatory ability for short-term survival prediction. This may be partly attributable to the small number of deaths occurring within the first year in the external validation cohorts. A nomogram integrating the risk classification with age, gender and tumor stage was further constructed to estimate individualized 1-, 3- and 5-year overall survival probabilities (Figure S4). Calibration curves showed generally good agreement between the predicted and observed survival probabilities, supporting the prognostic utility of the integrated model. These results support the seven-gene senescence-related signature as a complementary prognostic tool across multiple breast cancer cohorts.

### 3.5. Single-Cell and Spatial Analyses Identify Prognosis-Associated Cellular States Underlying the Senescence Signature

To elucidate the cellular context associated with the senescence-related prognostic signature, we integrated single-cell and spatial transcriptomic datasets to identify prognosis-associated cell populations and characterize their potential interactions within the tumor microenvironment. In the single-cell RNA-seq dataset, major cell populations were annotated, including epithelial cells, myeloid cells, plasmablasts, T cells, endothelial cells, CAFs and smooth muscle cells (Figure S5A). Marker gene expression and functional enrichment analyses supported the reliability of these annotations, with epithelial cells, CAFs, immune cells and vascular-associated cells showing distinct biological programmes consistent with their cellular identities (Figures S5B and S6). CopyKAT [42] analysis further distinguished aneuploid tumor cells from diploid non-malignant cells, and senescence-related pathway scoring showed that multiple senescence programmes were altered between tumor and normal cellular states, including cellular senescence, replicative senescence, DNA damage-induced senescence, oxidative stress-induced senescence and SASP-related signatures (Figure S5C–E). These results support the biological relevance of senescence-associated transcriptional states in breast cancer.

**Figure 5:**
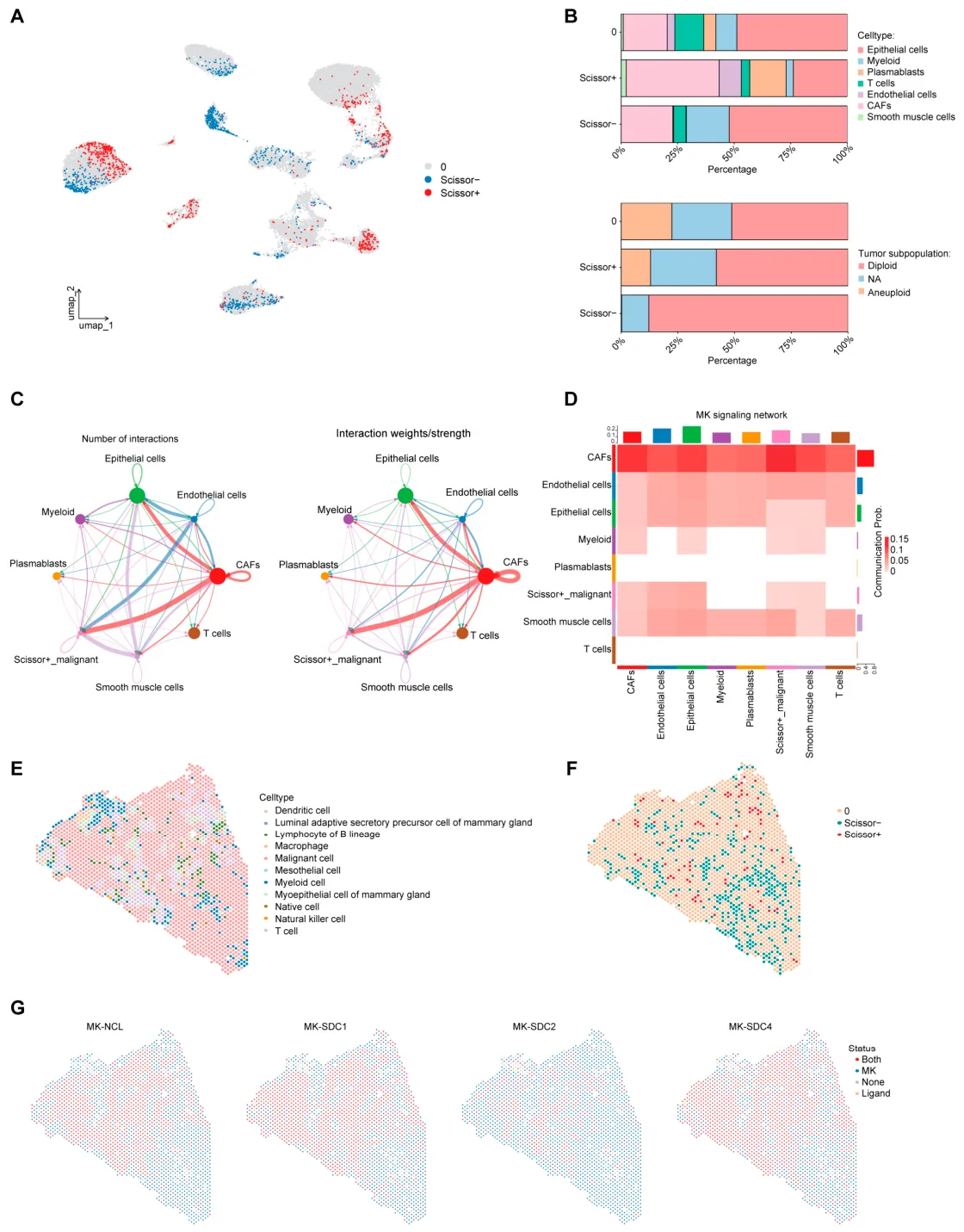
Identification and spatial validation of prognosis-associated cellular states. (**A**) UMAP visualization of Scissor analysis integrating bulk risk scores with single-cell RNA-seq data. Scissor+ cells were positively associated with the high-risk phenotype, whereas Scissor− cells showed the opposite association. (**B**) Cell-type composition and tumor subpopulation composition of Scissor+, Scissor− and background cells. (**C**) Cell–cell communication networks showing the number of interactions and interaction strength among Scissor+ malignant cells and other cell populations. Different colored edges indicate cell–cell communication interactions between different cell types. (**D**) Heatmap of the MK signalling network among Scissor+ malignant cells and tumor microenvironmental cell types. (**E**) Spatial transcriptomic mapping of major cell types in breast cancer tissue. (**F**) Spatial distribution of Scissor-defined cell states. (**G**) Spatial visualization of MK-related ligand–receptor communication pairs, including MK–NCL, MK–SDC1, MK–SDC2 and MK–SDC4.

To identify cell populations associated with bulk-level prognostic information, we applied Scissor [43] to integrate bulk and single-cell transcriptomic profiles using patient survival time and survival status as the phenotype. Scissor analysis identified Scissor+ cells positively associated with the high-risk phenotype and Scissor− cells showing the opposite tendency (Figure 5A). Cell composition analysis showed that Scissor+ cells were mainly distributed in malignant epithelial cells and several tumor microenvironment-related compartments, suggesting that the bulk risk score could be resolved into specific cellular states at single-cell resolution (Figure 5B).

We then examined the expression distribution of the seven signature genes in the single-cell landscape, with a particular focus on risk-associated genes with positive coefficients. LYVE1, HBEGF and ADGRF5 showed distinct but partially convergent cell-type distributions. LYVE1 was mainly detected in vascular/stromal-related populations, HBEGF was enriched in epithelial or tumor-associated cells, and ADGRF5 was preferentially expressed in CAFs, endothelial cells and smooth muscle cells (Figure S7). These findings suggest that the risk component of the prognostic signature reflects not only malignant epithelial cell states but also stromal–vascular remodelling in the tumor microenvironment [44,45].

Cell–cell communication analysis was further performed to explore potential interactions involving Scissor+ cells. Scissor+ malignant cells showed active communication with CAFs, endothelial cells and smooth muscle cells, with CAFs displaying prominent signalling activity (Figure 5C). Among the inferred communication pathways, the MK signalling network was one of the most notable axes. MK-related ligand–receptor pairs, including MK–NCL, MK–SDC1, MK–SDC2 and MK–SDC4, linked Scissor+ malignant cells with stromal and vascular cell populations (Figure 5D and Figure S8). Given the known involvement of MK signalling in tumor progression, migration, angiogenesis and microenvironmental remodelling [46,47], these results suggest that MK-mediated communication may contribute to the aggressive phenotype captured by the senescence-related risk model.

Spatial transcriptomic analysis was used to further validate the spatial organization of these prognosis-associated cellular states. Spatial mapping confirmed the presence of malignant cells and stromal/immune components within tumor tissues, and Scissor+ regions could be identified in spatial sections (Figure 5E,F). In addition, MK-related communication patterns were spatially observed in tumor regions, supporting the relevance of MK-mediated interactions within the tissue architecture (Figure 5G). Similar Scissor-associated distributions and MK communication features were observed in an independent spatial transcriptomic sample (Figure S9). Together, these results indicate that the senescence-related prognostic signature reflects a high-risk cellular ecosystem characterized by Scissor+ malignant cell states, stromal–vascular gene expression programmes and MK-mediated tumor–microenvironment communication.

### 3.6. ADGRF5 Promotes Breast Cancer Cell Migration and Invasion

**Figure 6:**
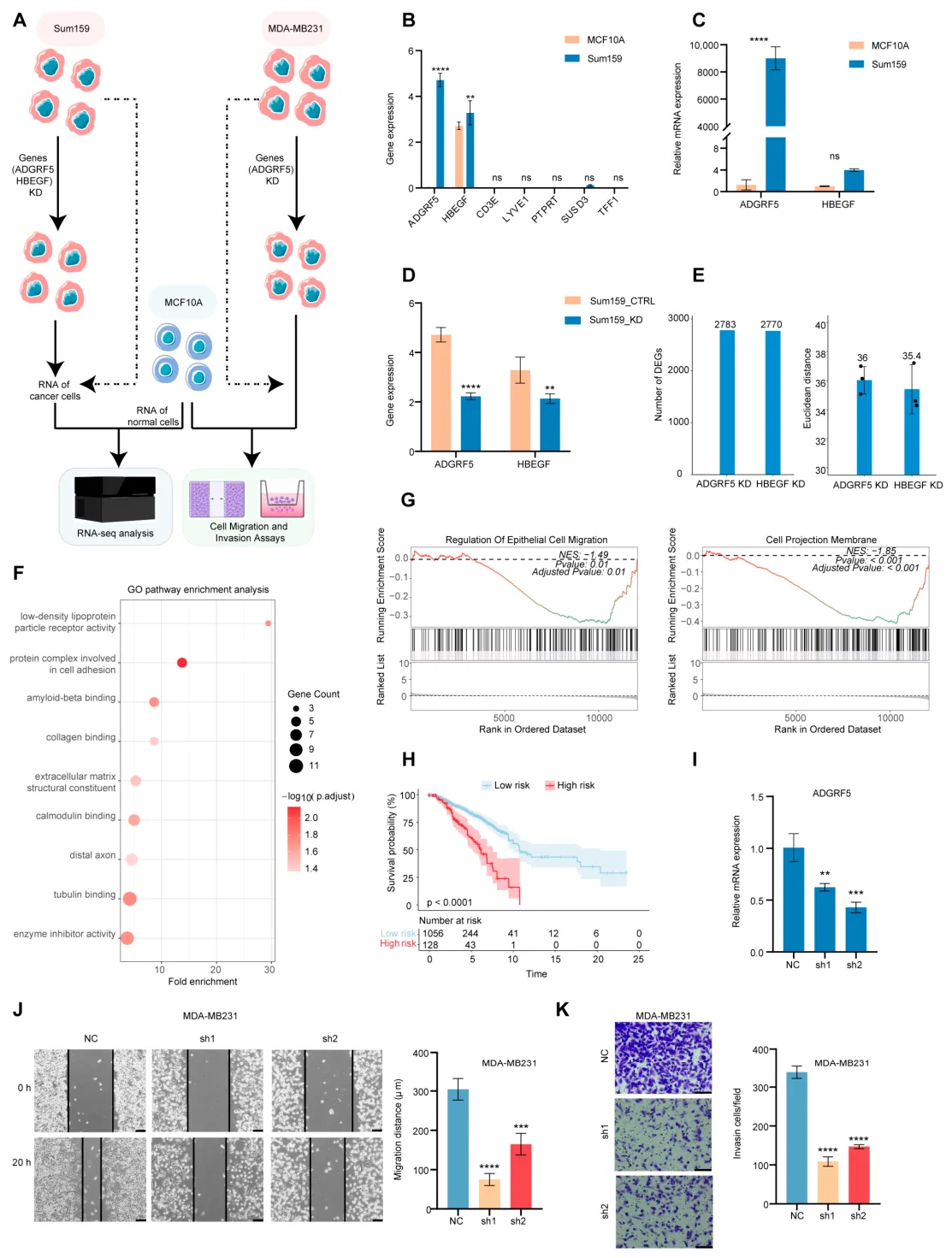
Experimental validation of ADGRF5 as a functional risk gene in breast cancer. (**A**) Schematic workflow of transcriptomic and functional validation. (**B**) Expression levels of the seven prognostic signature genes in SUM159 and MCF10A cells based on Smart-seq2 RNA sequencing. (**C**) qPCR validation of ADGRF5 and HBEGF expression in SUM159 and MCF10A cells. (**D**) Smart-seq2 RNA-seq analysis showing reduced ADGRF5 and HBEGF expression after gene knockdown in SUM159 cells. (**E**) Numbers of differentially expressed genes following ADGRF5 or HBEGF knockdown in SUM159 cells (left) and Euclidean distances between the transcriptomic profiles of knockdown and control samples (right). (**F**) GO enrichment analysis of ADGRF5-associated genes. (**G**) GSEA showing enrichment of ADGRF5-associated transcriptional programmes related to regulation of epithelial cell migration and cell projection membrane. The red and green lines represent the enrichment score curve, and the black vertical lines indicate the positions of genes in the ranked gene list. (**H**) Kaplan–Meier survival analysis of breast cancer patients stratified by ADGRF5 expression. (**I**) qPCR validation of ADGRF5 knockdown efficiency in MDA-MB-231 cells. (**J**) Wound-healing assay showing the effect of ADGRF5 knockdown on MDA-MB-231 cell migration. The dashed lines indicate the initial wound edges in the scratch assay. (**K**) Transwell invasion assay showing the effect of ADGRF5 knockdown on MDA-MB-231 cell invasion. ns, not significant; ** *p* < 0.01, *** *p* < 0.001, **** *p* < 0.0001.

To further prioritize functionally relevant genes from the senescence-related prognostic signature, we designed a validation workflow integrating transcriptomic profiling and functional assays (Figure 6A). SUM159 cells were used for Smart-seq2 RNA sequencing after ADGRF5 or HBEGF knockdown to evaluate transcriptomic alterations induced by candidate gene perturbation, whereas MDA-MB-231 cells were used for migration and invasion assays after ADGRF5 knockdown to further validate its functional role in an independent breast cancer cell context.

Smart-seq2 RNA sequencing showed that among the seven prognostic signature genes, ADGRF5 and HBEGF exhibited relatively greater expression differences between SUM159 and MCF10A cells, whereas the remaining genes showed limited changes (Figure 6B). Subsequent quantitative gene expression analysis showed that ADGRF5 was markedly and reproducibly upregulated in SUM159 cells, whereas the difference in HBEGF was less pronounced (Figure 6C). Together with their identification as prognosis-associated signature genes and their expression in malignant epithelial cells, these findings supported ADGRF5 and HBEGF as candidates for further functional investigation in breast cancer cells. Among them, ADGRF5 showed a more consistent differential expression pattern in the SUM159–MCF10A comparison and was therefore prioritized for subsequent investigation.

ADGRF5 and HBEGF were then knocked down in SUM159 cells, followed by Smart-seq2 RNA sequencing. The Smart-seq2 RNA-seq results showed that ADGRF5 and HBEGF expression levels were markedly reduced in the corresponding knockdown groups, confirming effective suppression of both genes (Figure 6D). ADGRF5 knockdown induced slightly more differentially expressed genes than HBEGF knockdown (2783 vs. 2770) and showed a marginally greater Euclidean distance from the SUM159 control (36.0 vs. 35.4), indicating a slightly greater transcriptomic perturbation following ADGRF5 depletion (Figure 6E). Therefore, ADGRF5 was selected for further characterization as a representative component of the senescence-related prognostic signature.

GO enrichment analysis of ADGRF5-associated genes revealed enrichment in cell adhesion, extracellular matrix structural components, tubulin binding and other migration-related functions (Figure 6F). GSEA further showed that ADGRF5-associated transcriptional changes were linked to regulation of epithelial cell migration and cell projection membrane pathways, suggesting a potential role of ADGRF5 in cytoskeletal organization and migratory regulation (Figure 6G). In addition, high ADGRF5 expression was associated with poorer survival in breast cancer patients, supporting its clinical relevance as a risk gene (Figure 6H).

To validate the functional role of ADGRF5, we performed loss-of-function assays in MDA-MB-231 cells. qPCR confirmed successful ADGRF5 knockdown using two independent shRNAs (Figure 6I). Wound-healing assays showed that ADGRF5 silencing significantly impaired cell migration (Figure 6J). Consistently, Transwell invasion assays demonstrated that ADGRF5 knockdown markedly reduced the invasive capacity of MDA-MB-231 cells (Figure 6K). To further validate these phenotypes in the same cellular context used for transcriptomic analysis, we additionally performed ADGRF5 knockdown in SUM159 cells. qPCR confirmed effective ADGRF5 suppression (Figure S10A), and both wound-healing and Transwell assays showed significantly reduced migration and invasion following ADGRF5 knockdown (Figure S10B,C). In addition, SA-β-gal staining showed an increased proportion of SA-β-gal-positive cells following ADGRF5 knockdown, providing preliminary evidence that ADGRF5 may be associated with a senescence-related phenotype (Figure S10D). Together, these results identify ADGRF5 as a key risk gene in the senescence-related prognostic signature and support its functional role in promoting breast cancer cell migration and invasion.

## 4. Discussion

Cellular senescence is a context-dependent biological programme with dual roles in tumour suppression and tumor progression. Although senescence can restrict the proliferation of damaged cells, senescent cells may also reshape the tumor microenvironment through inflammatory secretion, extracellular matrix remodelling, angiogenesis and immune regulation [48,49]. In breast cancer, the prognostic implication of senescence remains difficult to define because senescence lacks universal molecular markers and conventional literature-derived gene sets may not fully capture tumor-specific senescence states [11,50]. To address this limitation, we developed an AI-assisted framework to expand senescence-related gene discovery and established a senescence-related prognostic signature in breast cancer.

A key strength of this study is the use of single-cell foundation model-based in silico perturbation to identify senescence-associated genes beyond existing annotations. By modelling transcriptional transitions between young and old cellular states, Geneformer, scGPT and GeneCompass identified genes associated with rejuvenation-like or senescence-like shifts [21]. Compared with literature-derived senescence genes alone, AI-expanded gene sets showed improved prognostic stratification, with GeneCompass providing the strongest performance. The final candidate genes were enriched in inflammatory response, extracellular matrix organization, angiogenesis, epithelial differentiation and cell migration, suggesting that the AI-expanded signature captured biologically relevant senescence-associated programmes involved in breast cancer progression.

Based on these genes, we identified three senescence-related molecular subtypes with distinct clinical outcomes. Cluster 3 showed the poorest survival but had relatively low somatic mutation count, indicating that its adverse prognosis was unlikely to be driven simply by increased genomic mutation load [51]. Notably, Cluster 3 also exhibited the highest stromal score, whereas the highest immune score was observed in Cluster 2. This pattern raises the possibility that Cluster 3 may represent a stromal-rich, immune-excluded phenotype, in which senescence-associated stromal and extracellular matrix remodelling may restrict effective immune infiltration [52,53]. Instead, this subtype exhibited activation of multiple oncogenic and microenvironment-related pathways, including Hippo, Notch, PI3K, RAS, NRF2, TGF-β and Wnt signalling. Consistently, immune infiltration analysis revealed that Cluster 3 was characterized by increased M2 macrophage infiltration and higher angiogenesis and EMT scores. These findings suggest that the high-risk senescence subtype represents a transcriptionally and microenvironmentally remodelled tumor state associated with immune suppression, vascular remodelling and invasive progression [54]. Immune checkpoints are important regulators of tumor immune escape and immunotherapeutic response, with both classical and emerging checkpoint targets being increasingly explored in cancer treatment [55]. In this context, the M2 macrophage-enriched and pro-angiogenic phenotype observed in Cluster 3 may reflect an immunosuppressive tumor microenvironment, providing a broader immunotherapeutic context for this senescence-related subtype.

We further constructed a seven-gene prognostic model consisting of TFF1, SUSD3, PTPRT, LYVE1, HBEGF, CD3E and ADGRF5, which showed stable performance in the TCGA training cohort, an independent testing cohort and multiple external validation cohorts. Nevertheless, its discriminatory ability for short-term survival was relatively limited, as the 95% confidence interval of the pooled 1-year AUC included 0.5, potentially reflecting the small number of early survival events. Notably, this model contained both tumor epithelial-associated genes and microenvironment-related genes, indicating that the risk score reflects not only malignant cell-intrinsic features but also immune, stromal and vascular components. This multi-compartment nature may explain the robustness of the signature across heterogeneous breast cancer cohorts [45,56,57].

Multi-omics cancer research has increasingly highlighted the value of integrating complementary molecular data layers to better characterize tumor heterogeneity and the regulatory interactions underlying cancer progression [58]. In this context, single-cell and spatial transcriptomic analyses further clarified the cellular basis of the prognostic model. Scissor analysis mapped the bulk-derived high-risk phenotype to specific Scissor+ cellular states, mainly involving malignant epithelial cells and tumor microenvironment-associated compartments. Among the positive-risk genes, LYVE1, HBEGF and ADGRF5 showed distinct but complementary cellular distributions, linking the high-risk signature to stromal–vascular remodelling and malignant epithelial states. Cell–cell communication analysis further highlighted the MK signalling network, including MK–NCL, MK–SDC1, MK–SDC2 and MK–SDC4 interactions, as a potential communication axis between Scissor+ malignant cells and stromal/vascular cells. Spatial transcriptomic validation supported the tissue-level presence of these Scissor-associated cell states and MK-mediated interactions, suggesting that the prognostic signature reflects an organized high-risk tumor ecosystem [47,59] rather than isolated gene expression changes. The concordance among bulk, single-cell and spatial analyses supports the value of integrative approaches for resolving tumor complexity.

Among the seven model genes, ADGRF5 emerged as a prominent component of the senescence-related prognostic signature. Transcriptomic and qPCR analyses showed that ADGRF5 displayed more robust expression alteration than other candidate genes between breast cancer cells and non-tumorigenic mammary epithelial cells. ADGRF5 knockdown in SUM159 cells induced marked transcriptomic changes and showed stronger separation from control cells than HBEGF knockdown in PCA analysis. Functional enrichment and GSEA further linked ADGRF5-associated transcriptional programmes to cell adhesion, extracellular matrix components, cytoskeletal organization and epithelial cell migration [35,60]. Importantly, loss-of-function experiments in MDA-MB-231 cells confirmed that ADGRF5 silencing significantly impaired breast cancer cell migration and invasion. Together, these findings support ADGRF5 as a functionally relevant component of the senescence-related prognostic signature and provide additional biological support for its role within this prognostic framework.

Several limitations should be acknowledged. First, although the AI-assisted perturbation strategy expanded the senescence-related gene space, many predicted genes still require experimental validation. Second, the prognostic model was validated using multiple retrospective cohorts, but prospective clinical validation is needed before clinical application. Third, single-cell and spatial analyses revealed associations between high-risk cellular states, MK-mediated communication and stromal–vascular remodelling, but these findings remain correlative. Further mechanistic studies, including rescue assays and in vivo models, are required to determine whether ADGRF5 and MK-related interactions directly drive senescence-associated tumor progression.

In conclusion, this study establishes an AI-assisted senescence gene discovery framework and identifies a senescence-related prognostic signature with potential complementary value for breast cancer prognosis. By integrating bulk transcriptomics, machine learning, single-cell and spatial transcriptomics, and experimental validation, we revealed a high-risk senescence-associated tumor ecosystem characterized by oncogenic pathway activation, immune and stromal remodelling, MK-mediated intercellular communication and ADGRF5-driven migratory and invasive phenotypes. These findings provide new insight into the prognostic value of senescence-related cellular states and highlight ADGRF5 as a potential biomarker and functional target in aggressive breast cancer.

## Data Availability

The datasets analyzed in this study are publicly available from TCGA, GTEx, GEO and EMBL-EBI databases. TCGA-BRCA transcriptomic and clinical data were obtained from The Cancer Genome Atlas, and normal breast tissue data were obtained from the Genotype-Tissue Expression database. External validation datasets were downloaded from the Gene Expression Omnibus database, including GSE58644, GSE86166, GSE48390, GSE42568 and GSE199633. Single-cell RNA-seq datasets were obtained from E-MTAB-13664 and GSE180286, and spatial transcriptomic data were obtained from GSE203612. The Smart-seq2 RNA-sequencing data generated in this study from ADGRF5- and HBEGF-knockdown SUM159 cells have been deposited in the Science Data Bank (ScienceDB) and will be made publicly available upon publication of the article: https://www.scidb.cn/en/s/RvAZ7b (accessed on 18 August 2026). Other data supporting the findings of this study are included in the article and its Supplementary Materials.

## Supplementary Materials

The following supporting information can be downloaded at: https://www.mdpi.com/article/10.3390/cells15171589/s1, Figure S1: Comparison of prognostic stratification based on different senescence gene sets; Figure S2: Machine learning-based screening of prognostic genes from AI-expanded senescence-related candidate genes; Figure S3. Prognostic performance of the risk model in four independent cohorts; Figure S4: Construction and calibration of the integrated prognostic nomogram; Figure S5: Single-cell characterization of breast cancer cellular populations and senescence-related features; Figure S6: Marker gene expression of major cell types in breast cancer single-cell RNA-seq data; Figure S7: Single-cell expression distribution of the seven prognostic signature genes; Figure S8: MK signalling-associated cell–cell communication in single-cell RNA-seq data; Figure S9: Spatial validation of Scissor-associated cell states and MK-mediated communication in an independent spatial transcriptomic; Figure S10: Functional and senescence-related effects of ADGRF5 knockdown in SUM159 cells; Table S1: Primer sequences used for qPCR; Table S2: Multivariable Cox regression of the seven signature genes with hazard ratios reported per standard deviation.

## Abbreviations

The following abbreviations are used in this manuscript:

AI	Artificial intelligence
BC	Breast cancer
CS	Cellular senescence
KM	Kaplan–Meier
RF	Random forest
TCGA	The Cancer Genome Atlas
GEO	Gene Expression Omnibus
LSG	Literature-derived senescence-related genes
RG	Rejuvenation-associated genes
SG	Senescence-inducing genes
